# Changes in General Movements During Early Intensive Rehabilitation in High-Risk Infants with Structural Brain Injury: A Preliminary Study

**DOI:** 10.3390/children13050598

**Published:** 2026-04-27

**Authors:** Svetislav Polovina, Andrea Polovina, Jelena Erceg, Ema Dobrijević, Tanja Škorić Polovina, Romana Gjergja Juraški

**Affiliations:** 1 Polyclinic “Prof. dr. sc. Milena Stojčević Polovina”, Svetice 36, 10000 Zagreb, Croatia; info@poliklinika.org (S.P.); jelenaoreski@hotmail.com (J.E.); emakovacic170995@gmail.com (E.D.); skoric.polovina@gmail.com (T.Š.P.); romanagjergja68@gmail.com (R.G.J.); 2 Department of Endocrinology, University Hospital Centre Zagreb, Kišpatićeva 12, 10000 Zagreb, Croatia; 3 Neuropaediatric Department, Srebrnjak Children’s Hospital, Srebrnjak 100, 10000 Zagreb, Croatia; 4 Faculty of Medicine, Josip Juraj Strossmayer University of Osijek, J. Huttlera 4, 31000 Osijek, Croatia; 5 Faculty of Health Studies, University of Rijeka, Ulica Viktora cara Emina 5, 51000 Rijeka, Croatia

**Keywords:** early intervention, General Movements Assessment, cerebral palsy risk, cramped–synchronized, fidgety movements, high-risk infants, infant brain injury

## Abstract

**Highlights:**

**What are the main findings?**

- The Early Intensive Stojčević–Polovina Rehabilitation Method (EIR–SPM) was associated with a more optimal early motor repertoire.
- The Motor Optimality Score–Revised (MOS–R) increased between the first and second fidgety assessments during a period of intensive rehabilitation and ongoing neuroplasticity.

**What are the implications of the main findings?**

- Rehabilitation in high-risk infants can be initiated during the writhing period.
- In high-risk infants, rehabilitation can be achieved by using family settings.

**Abstract:**

**Background/Objectives:** Abnormal general movements (GMs) in high-risk infants are among the most sensitive early predictors of cerebral palsy (CP) and other neurodevelopmental disorders. This study described changes in the quality of GMs over time in high-risk infants who received the Early Intensive Stojčević–Polovina Rehabilitation Method (EIR–SPM). The EIR–SPM is a rehabilitation method designed for children with CP, those at risk of developing CP, and children with other developmental disabilities. In high-risk infants, it is initiated within the first three months of corrected age, preferably while writhing movements (WMs) are still present. **Methods:** This study was conducted in eight high-risk infants with abnormal WMs and structural brain injury. The EIR–SPM was initiated between 41 and 47 weeks postmenstrual age (PMA) and was applied until 60 weeks PMA. Prechtl’s General Movements Assessment (GMA), the Detailed GM score, and the Motor Optimality Score–Revised (MOS–R) were assessed. **Results:** During the writhing period, two infants showed a poor repertoire (PR) pattern and six showed a cramped–synchronized (CS) pattern of GMs; at follow-up, three showed PR, and five showed CS. During the fidgety period, two infants showed normal fidgety movements (F+), two sporadic fidgety movements (sFM), one infant showed abnormal fidgety movements (aFM), and three showed absent fidgety movements (F−) at the first assessment, while at the second assessment, three infants showed F+, two sFM, one aFM, and two F−. The median Detailed GM score increased from 12 (range 11–17) to 13.5 (range 11–19; *p* = 0.068). The median MOS–R increased from 17.0 (range 12–24) to 19.5 (range 17–27) between the two fidgety assessments (*p* = 0.027). **Conclusions:** Improvements in motor repertoire, reflected by increased MOS–R scores, were observed during the EIR–SPM initiated in the writhing period. Larger controlled studies are needed to confirm these preliminary observations.

## 1. Introduction

General movements (GMs) are the most common and complex patterns of spontaneous motor activity observed during fetal life and early infancy. GMs are age-dependent, variable sequences of arm, leg, neck, and trunk movements that involve the whole body. The General Movement Assessment (GMA) is a highly reliable tool that predicts various neurological and developmental disorders, particularly cerebral palsy (CP) [1], but also conditions such as mild developmental delay [2] or later motor coordination issues [3].

The prognostic value of GMA varies with the age at which the assessment is performed. From 46 to 49 weeks postmenstrual age (PMA), the character of GMs gradually changes from writhing movements (WMs) to fidgety movements (FMs) [1,4,5]. Injury to the central nervous system alters the quality of GMs and can lead to the appearance of abnormal GMs [6].

The most significant characteristic of abnormal GMs is the lack of complexity and variability of the quality of FMs. Absence of FMs has been found to be one of the most sensitive predictors of neurodevelopmental outcome in different populations of infants [1,7,8,9], and there is strong evidence that consistent abnormal WMs, especially a cramped–synchronized (CS) pattern, are highly predictive of later development of CP [10,11].

The interpretation of a poor repertoire (PR) pattern varies. Hadders–Algra [10] considers PR as a mild deviation from typical motor patterns, often reflecting a less severe or transient neurodevelopmental issue. In contrast, Einspieler [1] suggests that PR should not merely be considered a mild abnormality because the consistent presence of PR may, in exceptional cases, also lead to CP.

Early identification of high-risk infants is essential for the timely initiation of rehabilitation. Numerous studies provide evidence that early stimulation and an enriched environment can positively impact a developing nervous system, improving outcomes for high-risk infants [12,13,14,15,16,17,18]. Abnormal WMs have been identified as one of the earliest clinical markers of neurological dysfunction, particularly in infants with perinatal brain injury. While many studies have focused on the prognostic value of abnormal WMs [10,19,20,21], few have examined the potential of targeted early intervention to modify their quality [22,23,24,25,26,27].

To the best of our knowledge, the first study systematically investigating the effect of early rehabilitation on abnormal WMs in high-risk infants was conducted by Polovina (2011) [27]. The aim of the present study was to describe changes in the quality of GMs, MOS–R, and Detailed GM scores over time in high-risk infants with structural brain injury undergoing the EIR–SPM initiated during the writhing period.

## 2. Materials and Methods

### 2.1. Study Design

This was a single-center, preliminary study conducted at the Polyclinic “Prof. dr. sc. Milena Stojčević Polovina”, 10000 Zagreb, Croatia, with participant recruitment and data collection performed between June 2023 and June 2025. All consecutive infants who met the eligibility criteria and were referred for early rehabilitation were invited to participate.

This study included high-risk infants with abnormal GMs and confirmed structural brain injury, documented by cranial ultrasound and/or brain MRI. Infants were referred to the Polyclinic between 41 and 47 weeks PMA, and the first GMA was performed at the initial clinical visit.

GMA was conducted on four occasions in each infant: two assessments during the writhing period and two assessments during the fidgety period. WMs were assessed between 41 and 47 weeks (median 45 weeks at the first and 47 weeks at the second assessment), while FMs were assessed between 49 and 58 weeks PMA (median 52 weeks at the first and 55.5 weeks at the second assessment). According to the established GMA literature, WMs are typically observed from term age until approximately 46 to 49 weeks PMA, whereas FMs usually emerge from about 49 weeks PMA onwards; a brief transitional overlap between these periods may occur at the individual level [1,4,5]. Therefore, classification of movement patterns was based on the dominant spontaneous motor phenotype observed at the time of recording rather than on a strict chronological PMA threshold. The EIR–SPM was initiated during the writhing period, defined by the presence of WMs at the time of referral. Rehabilitation was initiated between 41 and 47 weeks PMA and was applied according to a standardized institutional protocol until 60 weeks PMA. The manuscript was prepared in accordance with the STROBE guidelines.

### 2.2. Participants

#### Eligibility Criteria

Inclusion criteria were the presence of abnormal WMs, defined according to the GMA as CS, PR, or chaotic (Ch) GMs; referral for rehabilitation during the writhing period; and structural brain injury confirmed by cranial ultrasound and/or brain MRI, extracted from clinical records. Exclusion criteria were normal WMs on GMA; absence of structural brain injury on cranial ultrasound and/or brain MRI; diagnoses not attributed to hypoxic–ischemic or vascular brain injury, including genetic, chromosomal, metabolic, or neuromuscular disorders; medical or non-medical inability to adhere to the EIR–SPM for at least two consecutive weeks; and contraindications for early intensive rehabilitation (e.g., severe congenital heart defects). All infants referred for early rehabilitation during the study period were screened for eligibility. A total of 59 infants were screened for eligibility during the study period. Eight infants met all inclusion criteria and were enrolled in the study. The remaining 51 infants were excluded due to ineligibility. Of the excluded infants, 12 exhibited normal WMs on GMA and were, therefore, not eligible. In 29 infants, structural brain injury could not be confirmed on cranial ultrasound and/or brain MRI. In seven infants, the underlying diagnosis was not attributed to hypoxic–ischemic or vascular brain injury, including one infant with brachial plexus palsy, one with spina bifida, three with congenital torticollis, and two infants with confirmed genetic disorders that were identified during clinical work-up. In addition, three infants were excluded due to an inability to adhere to the EIR–SPM protocol for at least two consecutive weeks. Each infant was excluded based on a single dominant criterion, with exclusion criteria applied hierarchically to avoid overlap between categories. No eligible infants declined participation. Given the narrow eligibility criteria, namely, the presence of abnormal WMs, confirmed structural brain injury, and referral during the writhing period, the number of eligible infants during the recruitment window was limited.

### 2.3. Neuroimaging and Classification of Structural Brain Injury

For the purposes of this study, structural brain injury was defined as a radiologically confirmed brain injury that was identified on cranial ultrasound and/or brain MRI, including intraventricular hemorrhage (Papile grade III or IV), periventricular leukomalacia (de Vries grade II or III), or hypoxic–ischemic injury patterns classified according to Barkovich et al. [28]. Structural brain injuries were classified by using established and validated systems based on the predominant type of injury observed. Intraventricular hemorrhage (IVH) was graded according to the Papile classification (Grades I–IV) [29]. Periventricular leukomalacia (PVL) was assessed using the system described by de Vries et al., including Grade I (increased periventricular echogenicity), Grade II (evolving cysts), and Grade III (established cystic lesions) [30]. Any hypoxic–ischemic encephalopathy (HIE) patterns observed on MRI were classified according to Barkovich et al. [28] into watershed, basal ganglia/thalamus, and total injury patterns. In some infants, lesions were considered not classifiable due to extensive malacia or atypical patterns that did not correspond to any established classification system. This multimodal approach was adopted to reflect the heterogeneity and complexity of brain injuries in the cohort.

### 2.4. Early Intensive Stojčević Polovina Rehabilitation Method (EIR–SPM)

The EIR–SPM is a rehabilitation approach designed for children with CP, children at risk of developing CP, and children with other developmental disabilities. The EIR–SPM has been routinely applied at the Polyclinic for many years as part of standard care for infants at high risk of CP and other neurodevelopmental disorders. In the present study, infants received the standard EIR–SPM protocol as part of the usual clinical care; no modifications were introduced for research purposes.

Rehabilitation was initiated at the institution, where parents received structured instruction and practical training. Institutional therapy sessions were conducted five times per week to monitor any clinical changes, guide parents, and adapt the program as needed. Concurrently, the EIR–SPM was continued at home, with the recommended frequency and duration adjusted according to the infant’s age, tolerance, and daily rhythm.

From approximately 40 to 44 weeks PMA, institutional therapy was scheduled five times per week, with each session lasting approximately 30 min. Each session included 15 min of therapist-led treatment and 15 min of parent-delivered therapy under a therapist’s supervision. During this phase, continuation of the EIR–SPM at home was recommended up to three times daily, with each home session lasting approximately 30 min. In addition to institutional therapy, families were instructed to deliver a minimum of 45 min of home-based EIR–SPM per day during this phase. Adherence to this minimum daily dose was monitored through structured verbal check-ins with parents at each institutional visit; these check-ins captured parental self-reporting and were not independently verified (e.g., time logs or diaries). Home-based doses were not recorded prospectively in minutes and were, therefore, not analyzed quantitatively.

Between 44 and 48 weeks PMA, institutional therapy continued five times per week, with the sessions lasting approximately 45 min, combining therapist-led rehabilitation and parental education. During this period, home-based therapy was recommended up to four times daily, with the sessions lasting approximately 30 min. The minimum required home-based dose in addition to institutional therapy was 60 min per day; adherence was monitored, as described above, based on parental self-reporting. From 48 to 60 weeks PMA, institutional therapy continued to be scheduled five times per week (45 min) and included therapist-led rehabilitation and parental education. During this phase, home-based therapy was recommended up to four times daily, with each session lasting approximately 45 min. The minimum required home-based dose in addition to institutional therapy was 90 min per day; adherence was monitored as described above, based on parental self-reporting.

The exact number of daily home therapy sessions was flexible; however, families were instructed to meet or exceed the minimum recommended daily home-based dose targets described above. The overarching therapeutic principle was to integrate the EIR–SPM into most cycles of the infant’s wakefulness, particularly following sleep and feeding. This approach was based on average age-appropriate wake–sleep patterns and clinical experience. Recommendations were, therefore, individualized and continuously adapted, with primary emphasis placed on the infant’s tolerance, behavioral state, and daily routine rather than on strict adherence to a fixed number of sessions.

For high-risk infants, rehabilitation was initiated during the writhing period. The intervention included positioning, physical therapy, and developmentally appropriate sensory stimulation. Infants were positioned in supine, prone, and lap positions. Supine and prone positions were selected according to the infant’s optimal developmental stage, defined as the developmental milestone at which the infant exhibited the least abnormal general movement patterns and the fewest abnormalities of muscle tone, as assessed using the GMA, clinical neurological examination, and evaluation of developmental milestones according to Vojta’s methodology [31]. This stage did not necessarily correspond to the infant’s chronological age. Intensive rehabilitation was conducted in these selected optimal developmental positions for an individually determined duration. Over time, positions were adjusted in accordance with changes observed in motor patterns, muscle tone, and development, following the trajectory of typical motor development described by Vojta [31].

Physical therapy included the following: (a) application of variable pressure to promote postural stabilization and control, combined with larger movements to enhance limb mobility; and (b) imitation of normal general movements. Imitation of general movements involved varying pressure combined with smooth and fluent movements of flexion, extension, and rotation. The speed, amplitude, and intensity of movements of the limbs, head, pelvis, shoulders, and trunk were systematically varied. Clear imitation of general movements was performed in the supine position, while in the lap and prone positions, more subtle, GM-like movements were also consistently elicited.

Throughout the EIR–SPM, particular attention was given to providing appropriate sensory input to support the development of visual and auditory functions. Visual stimulation included the use of high-contrast patterns, facial tracking, and object fixation tasks to promote oculomotor control and visual attention. Auditory stimulation included soft directional sounds, caregiver voice interaction, and environmental auditory cues to encourage sound localization and auditory orientation.

### 2.5. Video Recording and GM Assessment

Prechtl’s GMA was applied to evaluate the integrity of the central nervous system during the writhing and fidgety periods [1,4,5]. In addition to the qualitative classification of GMs, the assessment was complemented by the Detailed GM score and the MOS–R [1,32,33]. The Detailed GM score provides a structured quantitative assessment of GMs during the writhing period, focusing on movement complexity, variability, and fluency, with lower scores indicating a more severely impaired motor repertoire. The MOS–R is a composite score based on five subcategories—temporal organization of FMs, observed movement patterns, age-adequate movement repertoire, postural patterns, and movement character (i.e., an overall impression of movement quality)—with total scores ranging from 5 to 28, where higher scores indicate a more optimal motor repertoire. Infants were recorded between feedings during periods of active wakefulness, lying in the supine position with their wrists and ankles visible. All video recordings were independently assessed by three certified evaluators trained in Prechtl’s GMA. Two evaluators were not blinded to the infants’ identities or participation in the rehabilitation program due to the clinical setting. The third evaluator, who was not employed at the study institution, was blinded to all clinical information, neuroimaging findings, and the temporal order of the recordings. This external evaluator independently scored all recordings prior to any consensus discussion.

All raters were instructed to base their assessments exclusively on the observed movement quality by following standardized GMA criteria. Complete agreement among the evaluators was achieved for all assessments, except for one infant, which required a re-evaluation and consensus discussion; following reassessment, full agreement was achieved. Consensus ratings were used for the final analysis. Given the small sample size and the consensus-based final ratings, formal inter-rater reliability statistics (e.g., kappa or ICC) were not calculated. This is acknowledged as a methodological limitation. No computer-based algorithms were used for classification.

### 2.6. Outcomes and Statistical Analysis

Primary outcomes were the change in the MOS–R between the two fidgety assessments and the categorical GMA outcome during the fidgety period. The secondary outcome was the Detailed GM score during the writhing period. Categorical data were presented as absolute numbers and percentages, while numerical variables were reported as the median, minimum, and maximum. Paired comparisons refer to within-infant comparisons between the first and second assessments and were performed using the Wilcoxon signed-rank test.

All enrolled infants completed all planned assessments, and no missing data were present for the primary or secondary outcomes. Analyses were conducted using SPSS software (version 21).

## 3. Results

This study included four boys and four girls, with a median age of 45 weeks PMA (range 41–47), abnormal GMs, and a confirmed structural brain injury on neuroimaging (brain MRI and/or cranial ultrasound). A summary of the imaging characteristics and classifications is presented in Table 1. GMA was administered on four occasions (two during the writhing period; two during the fidgety period).

At the first writhing assessment, PR was observed in two infants (25%) and CS in six infants (75%). At the second writhing assessment, three infants (37.5%) exhibited PR, while five infants (62.5%) demonstrated CS. No infants with Ch were identified during the study period, and none exhibited normal WMs.

At the first fidgety assessment, two infants (25%) demonstrated normal fidgety movements (F+), two infants (25%) showed sporadic fidgety movements (sFM), one infant (12.5%) presented with abnormal fidgety movements (aFM), and three infants (37.5%) showed absent fidgety movements (F−). At the second fidgety assessment, three infants (37.5%) demonstrated F+, two infants (25%) showed sFM, one infant (12.5%) presented with aFM, and two infants (25%) showed F−. Changes in fidgety movement patterns were not uniform across the cohort. While some infants demonstrated improvement between the two fidgety assessments, others showed stable movement patterns. For example, one infant changed from F− at the first fidgety assessment to F+ at follow-up, whereas two infants remained classified as F− at both assessments. Overall, these findings indicate heterogeneous individual developmental trajectories within the cohort (Table 1).

### 3.1. Detailed GM Score

At the first assessment, the median Detailed GM score was 12 (range 11–17), while at the second assessment it increased to 13.5 (range 11–19; *p* = 0.068). Lower Detailed GM scores are typically observed in infants with markedly abnormal WMs [1]. At the first assessment, six infants (75%) had Detailed GM scores of 12 or lower, compared with four infants (50%) at the second assessment.

### 3.2. Motor Optimality Score–Revised (MOS–R)

At the first assessment, the median MOS–R was 17.0 (range 12–24). At the second assessment, the median MOS–R increased to 19.5 (range 17–27; *p* = 0.027). The median paired change in MOS–R scores between the first and second fidgety assessments was +2 points (range 0 to +12). Based on established cut-off criteria [32,33], six infants (75%) were classified as having a substantially reduced motor repertoire (≤19), no infants (0%) as non-optimal (20–23), and two infants (25%) as optimal (≥24) at the first assessment. At the second assessment, four infants (50%) remained in the substantially reduced category, one infant (12.5%) was classified as non-optimal, and three infants (37.5%) achieved optimal MOS–R scores.

## 4. Discussion

This study explored changes in the quality of GMs in a small cohort of high-risk infants who underwent the EIR–SPM during the writhing and fidgety periods. The findings provide insight into how early motor patterns may evolve in infants with severe structural brain injury when intensive rehabilitation is initiated early in development. In addition, this study highlights the value of combining qualitative and quantitative GMA-based measures to characterize early neurodevelopmental vulnerability.

In observational studies of this type, it is difficult to distinguish the effects of rehabilitation from spontaneous developmental changes related to intrinsic neuroplasticity. To minimize the likelihood that the observed changes reflected spontaneous maturation alone, the present study applied restrictive inclusion criteria and focused exclusively on infants at very high risk of persistent neurological impairment. All included infants demonstrated a convergence of markedly abnormal WMs, predominantly CS, low Detailed GM scores, and a confirmed structural brain injury on cranial ultrasound and/or brain MRI.

Abnormal WMs and low Detailed GM scores indicate reduced movement complexity and variability; together with confirmed structural brain injury, these features have been consistently associated with adverse neurodevelopmental outcomes. Specifically, Ferrari et al. demonstrated that consistent or predominant CS general movements were followed by cerebral palsy in all affected infants, with a specificity of 92.5% to 100% across the observed age range, and none of these infants exhibited fidgety movements at follow-up [11]. These findings suggest that spontaneous normalization in infants with consistent CS and confirmed structural brain injury is unlikely, which reduces—though does not eliminate—the probability that the observed improvements in MOS–R scores in the present cohort reflect developmental maturation alone. As this is a preliminary study, future research should include a control group to address the potential impact of developmental maturation.

In the present study, improvements in MOS–R scores during the fidgety period were observed in some high-risk infants. The observed median increase of 2 points in MOS–R between assessments may be clinically meaningful, as recent evidence suggests that a trajectory of improvement of ≥2 points on the MOS–R is associated with significantly higher odds of scoring optimally on the Hammersmith Infant Neurological Examination (HINE) [34]. Nevertheless, developmental maturation and other non-intervention factors cannot be excluded as potential explanations for the observed changes.

The type of intervention and the timing of its initiation were key aspects of this study. The EIR–SPM is a multicomponent, highly intensive intervention aimed at modulating the infant’s spontaneous motor activity. In the EIR–SPM, this goal is addressed through the techniques described above, among which a key component is the imitation of normal GMs in the supine position, which systematically integrates movements of the limbs, trunk, and head with appropriate postural control. This approach is based on the assumption that eliciting more typical patterns of spontaneous motor activity may support the emergence of a more optimal early motor repertoire.

Spontaneous motor activity in typically developing infants is not restricted to the supine position and can also be observed in the prone position. Prechtl described GMs, particularly FMs, as an age-specific fine-tuning of the proprioceptive system [1], and within the EIR–SPM, we adopted the rationale that this process is not necessarily restricted to the supine position. Accordingly, in addition to the imitation of normal GMs in the supine position, the EIR–SPM also incorporates the imitation of GM-like movement patterns in the prone position and while the infant is held in the caregiver’s arms, although such movement patterns have not been formally described within the framework of the GMA.

Another key element of the EIR–SPM is the individualized selection of optimal developmental positions. Within these positions, more typical patterns of spontaneous motor activity may be elicited. Within this favorable motor repertoire, the infant may generate spontaneous movements that more closely resemble those observed in typically developing infants, which could contribute to changes in the observed quality of GMs despite the fact that GMs are considered to be endogenously generated and only minimally influenced by environmental stimulation [1]. The timing of rehabilitation initiation represents a critical aspect of the EIR–SPM. The intervention is initiated during the writhing period, which coincides with a phase of heightened experience-dependent brain plasticity.

Early infancy is characterized by rapid synaptogenesis and the large-scale reorganization of neural circuits involved in motor control [35,36]. This developmental window overlaps with the transition from WMs to FMs, reflecting increasing cortical modulation of spontaneous motor activity [37,38,39]. Initiating rehabilitation while WMs are still present may, therefore, provide enriched sensorimotor input at a time when developing motor networks are particularly receptive to environmental influences [40]. This period coincides with the regression of the transient subplate zone and the establishment of permanent neural circuits in the primary motor cortex [37,38], representing a pivotal ontogenetic window during which experience-dependent plasticity is particularly prominent. These neurobiological considerations support the rationale for initiating the EIR–SPM during the writhing period, regardless of whether the infant was born at term or preterm.

In the present study, improvements in the MOS–R scores during the fidgety period were observed at the group level, although individual developmental trajectories remained heterogeneous. These findings do not challenge the established prognostic value of the GMA or the MOS–R [32,33]. Instead, these findings suggest that early motor assessments reflect ongoing developmental changes and, therefore, need to be interpreted over time, especially during early infancy when development is rapid. Notably, while some infants achieved optimal MOS–R scores at follow-up, a substantial proportion remained within the non-optimal range, which is clinically relevant and warrants continued developmental surveillance and rehabilitation. Non-optimal MOS–R outcomes have been associated not only with later motor impairment but also with cognitive and behavioral difficulties in childhood [41,42].

The idea of initiating rehabilitation in early infancy can be traced back to the work of Stojčević Polovina, who, already in 1978, identified the first three months of corrected age as a critical period for starting rehabilitation in high-risk infants [43]. This clinically derived observation aligns temporally with contemporary neurodevelopmental concepts and the developmental transition from WMs to FMs described in the GMA literature. To the best of our knowledge, the first study systematically investigating the effect of early rehabilitation on abnormal WMs in high-risk infants was conducted by Polovina (2011) [27]. In that study, the impact of early intensive rehabilitation was investigated in a cohort of 31 high-risk infants with abnormal WMs. The results showed that infants with PR who participated in the early intensive rehabilitation exhibited a significantly higher occurrence of FMs compared with the control group, whereas no significant difference was observed between the two groups of infants with CS.

Among existing early intervention approaches, Movement Imitation Therapy for Preterm Babies (MIT–PB), as described by Soloveichick et al. [25], represents the most conceptually comparable intervention to the EIR–SPM, as it explicitly targets the quality of GMs. In their pilot study, Soloveichick et al. described four very preterm infants, three with grade III intraventricular hemorrhage and one with intraventricular hemorrhage with apparent periventricular hemorrhagic infarction. All infants exhibited cramped–synchronized (CS) general movements at 33–35 weeks PMA, and MIT–PB was initiated shortly thereafter.

Despite these differences in intervention design, the outcomes reported in the study by Soloveichick et al. and in the present cohort appear conceptually aligned [25]. Similar observations have been reported in a larger observational study by Toma et al., although infants in that cohort generally presented with lower neurodevelopmental risk [44]. Together, these findings support the notion that early, targeted, and sufficiently intensive interventions initiated during sensitive developmental periods may be associated with more optimal early motor repertoire development in some high-risk infants.

Several limitations should be acknowledged. The small sample size and absence of a control group limit the statistical power and generalizability of the findings, and it cannot be ruled out that the observed changes partly reflect natural developmental maturation rather than the effects of the intervention. Adherence to the home-based component relied on parental self-report collected through structured verbal check-ins at each of the five weekly institutional visits, at which all families confirmed that the minimum recommended daily dose had been met. While the high frequency of institutional contact provided repeated opportunities to monitor adherence, and parental technique was continuously assessed during supervised practice within sessions, independent objective monitoring was not performed, and variability in the actual dose delivered between families cannot be excluded. Future research should prospectively quantify home program doses using standardized logs or objective monitoring tools. Furthermore, the lack of blinding of two evaluators represents a potential source of observer bias that cannot be excluded, and although consensus was reached in all cases, this limitation should be addressed in future studies through fully blinded assessment procedures. As the EIR–SPM is a multicomponent intervention, the relative contribution of individual therapeutic elements cannot be determined from this study design. Finally, the heterogeneity of brain injury types reflects real-world clinical populations but limits conclusions regarding specific lesion patterns. Despite these limitations, the current findings provide valuable preliminary evidence supporting the potential of the EIR–SPM in high-risk infants.

## 5. Conclusions

The present findings suggest that changes in early motor patterns can occur over relatively short developmental intervals in high-risk infants, potentially reflecting developmental change in early infancy during a period of intensive rehabilitation and ongoing neuroplasticity.

Improvements in motor patterns likely reflect the combined influence of the EIR-SPM and intrinsic neuroplasticity. Even in high-risk infants with severe brain injury, enrolment in the EIR-SPM during the writhing period—which may be considered a critical stage of early development—may promote more favorable neurodevelopmental outcomes. The only feasible way to achieve the intensity required by the EIR-SPM during these critical developmental stages is through a home-based, family-centered approach, in which trained parents provide rehabilitation under regular therapist supervision. These results represent preliminary findings, and larger controlled studies are needed to further examine the effects of early, developmentally timed rehabilitation in high-risk infant populations.

## Figures and Tables

**Table 1:** Main imaging findings and motor repertoire.

Infant	Main imaging Findings	Papile Grade	PVL Grade (de Vries)	HIE Pattern (Barkovich)	GMA (41–47 Weeks PMA) 1st	GMA (41–47 Weeks PMA) 2nd	GMA (49–58 Weeks PMA) 1st	GMA (49–58 Weeks PMA) 2nd
1	Post-hemorrhagic hydrocephalus; frontotemporal parenchymal malacia; very thin cortex	Grade IV	Grade III	Not classifiable (extensive malacia)	PR	PR	F+	F+
2	Left porencephalic cyst (1.6 cm); asymmetric ventricles	–	Grade III	Likely watershed pattern	PR	PR	sFM	sFM
3	Dilated lateral ventricles; no acute hemorrhage	–	Grade II	Not classifiable	CS	CS	F−	F+
4	MRI: bilateral cortical and subcortical lesions	–	–	Watershed type	CS	CS	F+	F+
5	MRI: thalamic and lateral geniculate body lesions	–	–	Basal ganglia/thalamus type	CS	PR	aFM	aFM
6	MRI: diffuse cortical injury	–	–	Watershed type	CS	CS	sFM	sFM
7	Frontal PVL; mild ventricular dilatation	Grade III	Grade II–III	PVL pattern	CS	CS	F−	F−
8	Post-hemorrhagic hydrocephalus	Grade III	–	Not classifiable	CS	CS	F−	F−

Abbreviations: PR, poor repertoire; CS, cramped–synchronized; F+, normal fidgety movements; F−, absent fidgety movements; sFM, sporadic fidgety movements; aFM, abnormal fidgety movements; “–”, not applicable or not available, HIE, hypoxic–ischemic encephalopathy; PVL, periventricular leukomalacia; MRI, magnetic resonance imaging.

## Data Availability

Anonymized numerical data and anonymized clinical findings used in this study may be made available from the corresponding author upon reasonable request and following approval by the institutional ethics committee. Access to video recordings of infants could also be considered; however, due to their sensitive nature, this would additionally require written parental consent and ethics committee approval.

## Abbreviations

The following abbreviations are used in this manuscript:

GMs	General movements
CP	Cerebral palsy
PMA	Postmenstrual age
EIR–SPM	Early Intensive Stojčević–Polovina Rehabilitation Method
GMA	Prechtl’s General Movements Assessment
MOS–R	Motor Optimality Score–Revised
WMs	Writhing movements
CS	Cramped–synchronized
PR	Poor repertoire
Ch	Chaotic
FMs	Fidgety movements
F+	Normal fidgety movements
sFM	Sporadic fidgety movements
aFM	Abnormal fidgety movements
F−	Absent fidgety movements
HINE	Hammersmith Infant Neurological Examination
MRI	Magnetic resonance imaging
IVH	Intraventricular hemorrhage
PVL	Periventricular leukomalacia
HIE	Hypoxic–ischemic encephalopathy
ICC	Intraclass correlation coefficient
